# Global trends in the proportion of macrolide-resistant *Mycobacterium* Species: A systematic review and meta-analysis

**DOI:** 10.1371/journal.pone.0333521

**Published:** 2025-11-07

**Authors:** Saeid Sadeghi Ghazi Chaki, Saeed Babaie Pirsoltan, Masoumeh Beig, Tahereh Navidifar, Elnaz Parvizi, Maryam Mofid, Narges Golab, Mohammad Sholeh

**Affiliations:** 1 Department of Microbial Biotechnology, School of Biology and Center of Excellence in Phylogeny of Living Organisms, College of Science, University of Tehran, Tehran, Iran; 2 Department of Biology, Faculty of Convergent Science and Technology, Sciences and Research Branch, Islamic. Azad University, Rasht, Iran; 3 Department of Bacteriology, Pasteur Institute of Iran, Tehran, Iran; 4 Department of Basic Sciences, Shoushtar Faculty of Medical Sciences, Shoushtar, Iran; 5 Department of Microbiology, Science and Research Branch, Islamic Azad University, Tehran, Iran; 6 School of Medicine, Hamadan University of Medical Science, Hamadan, Iran; 7 Department of Microbiology, School of Medicine, Tehran University of Medical Sciences, Tehran, Iran; Lady Hardinge Medical College, INDIA

## Abstract

**Background:**

Macrolide resistance in *Mycobacterium* species is an emerging global problem, complicating treating non-tuberculous mycobacteria (NTM) and *Mycobacterium tuberculosis*. Understanding global resistance patterns is essential to improve treatment strategies and reduce morbidity and mortality.

**Objective:**

This study aimed to investigate macrolide resistance prevalence, distribution, and trends among *Mycobacterium* species in different world regions.

**Methods:**

A comprehensive search of Scopus, PubMed, Web of Science, and EMBASE (2015–2023) assessed study quality using JBI guidelines. Resistance proportions were calculated with a random-effects model, and additional meta-regression and subgroup analyses explored variations and trends. Statistical analyses were conducted using R and the metafor package.

**Results:**

Out of 5,718 records, 41 studies across 17 countries were included. Azithromycin resistance was reported in 17 reports (3 Studies), with 156 resistant isolates among 715 tested, yielding a pooled rate of 29.0% (95% CI: 19.5–40.7%). Clarithromycin resistance, assessed in 76 reports (40 Studies), included 1,071 resistant isolates among 3,923 tested, with a pooled rate of 30.5% (95% CI: 23.7–38.2%). Erythromycin resistance was reported in 17 reports (3 Studies), with 350 resistant isolates among 752 tested, giving a pooled rate of 50.3% (95% CI: 37.5–62.9%). Meta-regression revealed a significant upward trend in azithromycin resistance over time (r = 0.558, p = 0.005).

**Conclusion:**

These findings underscore the need for targeted, region- and species-specific interventions to combat rising macrolide resistance in *Mycobacterium* species effectively.

## 1. Introduction

*Mycobacterium* species, particularly *Mycobacterium tuberculosis* (TB) and various nontuberculous mycobacteria (NTM), present significant global health challenges due to their association with severe infections, such as tuberculosis and chronic pulmonary diseases [1,2].

These infections predominantly affect individuals with compromised immune systems, leading to increased morbidity and mortality rates [3].

The treatment of these infections often relies on macrolides such as clarithromycin and azithromycin, which inhibit bacterial protein synthesis [4].

However, the emergence of macrolide-resistant strains poses a formidable challenge in managing these infections, complicating treatment protocols and outcomes [5].

The prevalence of macrolide resistance in *Mycobacterium* species is a growing concern. This is exacerbated by the extensive use of these antibiotics in clinical settings, agriculture, and aquaculture, creating significant selection pressure [6].

Understanding the distribution and prevalence of macrolide resistance across different *Mycobacterium* species and geographical regions is critical for developing effective treatment strategies [7–10]. Despite numerous studies exploring this resistance, a notable gap remains in comprehensive data synthesis, particularly regarding global and regional resistance trends.

Many existing studies are limited to specific locales, which hinders a broader understanding of the resistance patterns and their temporal evolution [11].

Moreover, the methodologies employed in antimicrobial susceptibility testing (AST) vary significantly across studies, leading to inconsistent results that complicate comparisons and meta-analyses [12].

Most existing studies have concentrated on a limited subset of *Mycobacterium* species, often overlooking the broader diversity of NTMs that may exhibit significant macrolide resistance [13]. Methodological inconsistencies among studies may also introduce bias in estimating resistance prevalence [14]. Moreover, there is a critical need for detailed subgroup analyses based on species, geographic regions, and patient demographics to better characterize resistance patterns [15].

To address these gaps, our systematic review and meta-analysis aimed to comprehensively assess macrolide resistance in *Mycobacterium* species. Specifically, we estimated resistance prevalence across regions evaluated temporal trends and assessed how different AST methods influence reported resistance rates [12]. We also examined species-specific resistance, particularly in underrepresented NTMs, and analyzed study quality to evaluate its impact on reported findings [15]. Through subgroup analyses stratified by geography, time, species, AST methods, and study quality, our synthesis offers in-depth insights to inform clinical decision-making and guide public health policies on antibiotic resistance.

## 2. Methods

Our study meticulously followed the Preferred Reporting Items for Systematic Reviews and Meta-Analyses (PRISMA) guidelines [16] to ensure a comprehensive and reliable synthesis of data on macrolide resistance in *Mycobacterium* species. Additionally, our registration in the PROSPERO database (CRD42024565676) reflects our commitment to transparency and adherence to rigorous methodological standards.

### 2.1. Eligibility criteria

To ensure the rigor and relevance of the synthesized evidence, we applied clearly defined inclusion and exclusion criteria. Eligible studies investigated macrolide resistance in *Mycobacterium* species and reported both resistance proportions and corresponding sample sizes. Only peer-reviewed articles published in English and available in full text were considered. The restriction to English-language publications was applied to ensure consistent data interpretation and avoid inaccuracies that may arise from translation.

Studies were excluded if they were case reports, narrative reviews, single-arm studies, pharmacokinetic studies, or clinical trials, as these designs typically lack generalizable prevalence data or standardized AST methods necessary for meta-analysis. Furthermore, studies involving fewer than three isolates or reporting antibiotic susceptibility based on fewer than three isolates were excluded to reduce the potential for statistical bias and ensure meaningful estimates of resistance. These criteria were selected to maintain methodological consistency, minimize heterogeneity, and enhance the validity and reproducibility of the meta-analytic results.

#### 2.1.1. Included *Mycobacterium* Sub-species.

This review focused on macrolide resistance in multiple Mycobacterium sub-species. The included sub-species, as extracted from eligible studies, were *M. abscessus*, *M. avium*, *M. massiliense*, *M. bolletii*, *M. chelonae*, *M. fortuitum*, *M. gordonae*, *M. intracellulare*. These sub-species were identified either explicitly in the included articles or inferred based on context, and all were analyzed in subgroup analyses of resistance prevalence.

### 2.2. Information sources and search strategy

A thorough systematic search of online databases (Scopus, PubMed, Web of Science, and EMBASE) was conducted up to December 2023 to identify all relevant studies. The search strategies for each database were tailored according to specific guidelines, as detailed in the Supplementary File.

### 2.3. Selection process

After importing the results of a systematic online database search into EndNote (version 20) and removing duplicates, two authors (T-N and N-G) independently reviewed and analyzed the relevant publications to ensure an unbiased process. A third author (M-B) resolved any discrepancies between their analyses, evaluated the differences, and made the final decision.

### 2.4. Data collection process

To ensure accuracy, two authors (M-M, E-P, and SBP) independently collected the data and resolved discrepancies through mutual agreement.

#### 2.4.1. Data Items.

The extracted data included the names of the first authors, the year of publication, the countries involved, the diagnostic methods used, the sample sources, the number of positive tests, and the overall sample size.

### 2.5. Study risk of bias assessment

The quality of the included articles was assessed using the Joanna Briggs Institute (JBI) criteria Field (10). Two authors (M-B and M-M) independently performed this evaluation. A third author (M-SH) resolved the discrepancies between assessments, reviewed the articles, and made the final decision.

### 2.6. Effect measures

This meta-analysis assessed the prevalence of antibiotic resistance by analyzing the proportion of resistant isolates reported in multiple studies. Subgroup and meta-regression analyses were employed to identify factors contributing to variations in resistance rates, including geographic origin and other relevant variables. Additionally, the study evaluated temporal trends in macrolide resistance to understand the changes over time.

### 2.7. Synthesis methods

We conducted a meta-analysis using a random-effects model, with between-study heterogeneity (τ²) estimated via the DerSimonian–Laird method. Heterogeneity was quantified using the Q-test and the I² statistic; any indication of heterogeneity (τ² > 0) was explored further, regardless of Q-test significance. To examine temporal trends in antibiotic resistance, we performed meta-regression analyses. Outlier and influence diagnostics were conducted using studentized residuals and Cook’s distances, respectively. Specifically, studies with studentized residuals exceeding the Bonferroni-adjusted 100×(1–0.05/2k) percentile of a standard normal distribution were considered outliers, while those with Cook’s distances above the median plus six times the interquartile range were flagged as influential.

Publication bias was assessed through funnel plot asymmetry tests, including both rank correlation and regression methods, using the standard error of observed outcomes as predictors. In addition, we applied the DOI (Deviation from Optimal Intervention) plot with the Luis Furuya-Kanamori (LFK) index to further evaluate small-study effects. The DOI plot provides a more sensitive visualization of asymmetry compared to the conventional funnel plot, while the LFK index offers a quantitative measure: values within ±1 indicate no asymmetry, values between ±1 and ±2 suggest minor asymmetry, and values beyond ±2 indicate major asymmetry.

All statistical analyses were conducted in R (version 4.2.1) using the metafor package (version 3.8.1) [17–24].

### 2.8. Reporting bias assessment and certainty assessment

Using the rank correlation and Egger’s regression tests, we assessed funnel plot asymmetry and potential reporting bias. To further explore and visualize small-study effects, we also generated DOI plots with the Luis Furuya-Kanamori (LFK) index, which provide a more sensitive graphical method for detecting asymmetry compared to traditional funnel plots. An LFK index within ±1 suggests no asymmetry, values between ±1 and ±2 indicate minor asymmetry, and values beyond ±2 suggest major asymmetry. In addition, Fail-Safe N and Trim-and-Fill methods were applied to estimate the potential impact of unpublished studies and to adjust for missing data, thereby enhancing the robustness and credibility of our findings.

## 3. Results

### 3.1. Study selection

**Fig 1:**
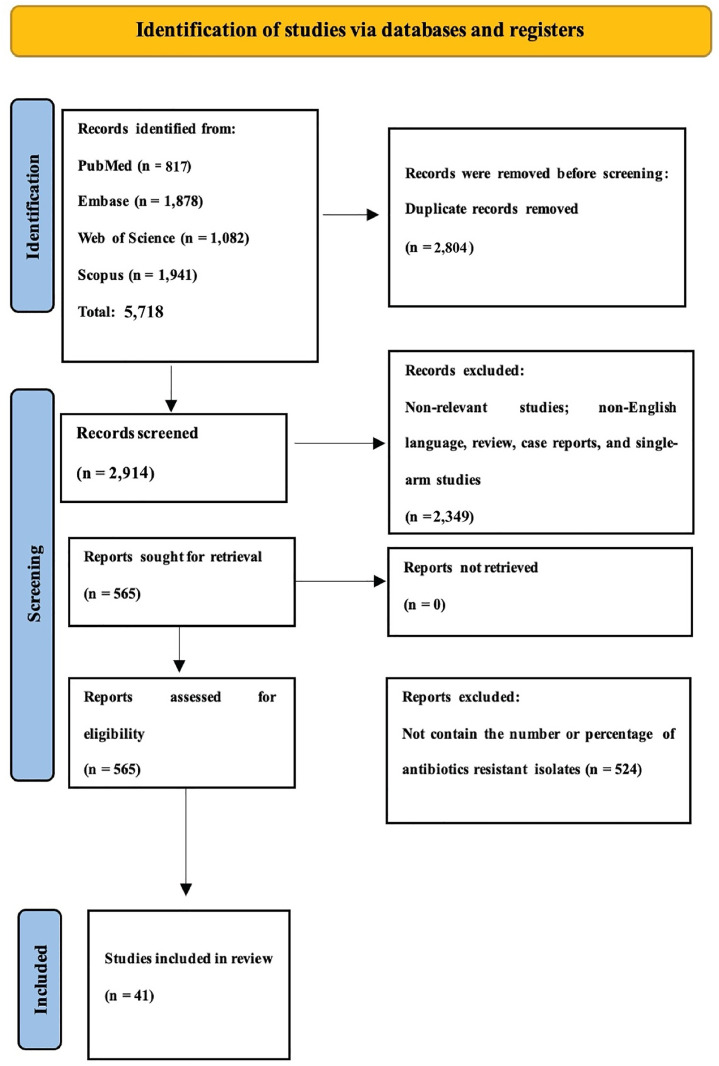
PRISMA flow chart summers the article selection procedure. This PRISMA flow diagram illustrates identifying and selecting relevant studies for inclusion in the review. Studies were identified through a comprehensive search of PubMed, Embase, Web of Science, and Scopus databases. After removing duplicates and excluding irrelevant studies, 37 were included in the final review.

In this systematic review and meta-analysis, a comprehensive search yielded 5,718 records managed using EndNote version 20 software. After removing 2,804 duplicates, 2,914 articles were retained for title and abstract screening. Of these, 565 full-text articles were evaluated, including 37 studies that met the criteria [14, 25–60]. Additional supplementary material, including extracted datasets, subgroup analyses, and extended methodological details, is provided in the S1 File. Details characteristics of the included studies, along with the extracted data, including first author, year, country, study design, sample size, and macrolide-resistance data, are provided in S2 File. The PRISMA flowchart summarizes the screening and selection processes (Fig 1 and S3 File).

### 3.2. Study characteristics

Between 2015 and 2023, 41 studies were included in this meta-analysis, originated from Asia (Japan, 9; China, 9; Korea, 3; South Korea, 1; Iran, 2; Singapore, 2; Taiwan, 2; Malaysia, 1; Thailand, 1; India, 1; Lebanon, 1), the Americas (USA, 4; Brazil, 1), and Europe (Germany, 1; Portugal, 1; Spain, 1), with one study not reporting its country of origin. In total, 32 studies were from Asia, 5 from the Americas, and 3 from Europe.

Study quality was assessed using the JBI critical appraisal checklist. Of the 41 studies, 40 were rated as low risk of bias, while one was rated as having some risk. Chronologically, 17 studies were conducted between 2015 and 2018, and 24 between 2019 and 2023.

Antimicrobial susceptibility testing (AST) methods varied, including minimum inhibitory concentration (MIC) testing (n = 15), multiple methods (n = 19), automated systems (n = 3), disk diffusion (n = 2), and two studies that did not specify the method used.

### 3.3. Comprehensive overview of antibiotic resistance prevalence

#### 3.3.1. Prevalence of Azithromycin resistance.

**Fig 2:**
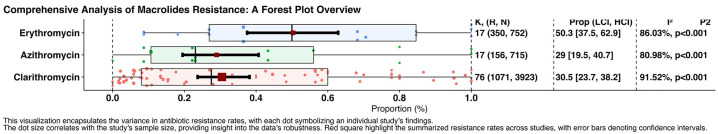
Forest plot summarizing the prevalence of antibiotic resistance in individual studies. Each dot represents the resistance rate in a single study, with the size of the dot reflecting the sample size. The red square and error bars indicate the pooled estimate and 95% confidence interval.

In our analysis of azithromycin resistance, we included data on 715 isolates from 17 reports (3 Studies). Using a random-effects model, we estimated an average resistance proportion of 0.290 (95%CI, 0.195, 0.407). Therefore, the average outcome differed significantly from zero (z = −3.366, p < 0.001, Table 1). According to the Q-test, the true outcomes appear to be heterogeneous Q(16) = 84.113, I² = 80.98%, p < 0.001. A forest plot showing the observed outcomes and the estimate based on the random-effects model is shown in Fig 2. With the fill and trim method implementation the proportion changed to 0.290 (95%CI, 0.195, 0.407, Table 2). An examination of the studentized residuals revealed that none of the studies had a value larger than 2.974 and hence there was no indication of outliers in the context of this model. According to the Cook’s distances, none of the studies could be considered to be overly influential. The regression test indicated funnel plot asymmetry (p = 0.019) but not the rank correlation test (p = 0.095).

**Table 1:** Meta-analysis statistics of antibiotic-resistant *Mycobacterium* species worldwide.

Antibiotic	K (n, N)	Proportion 95%CI(LCI, HCI)	I²	P1
Azithromycin	17 (156, 715)	0.290 (0.195, 0.407)	80.98%	p < 0.001
Clarithromycin	76 (1071, 3923)	0.305 (0.237, 0.382)	91.52%	p < 0.001
Erythromycin	17 (350, 752)	0.503 (0.375, 0.629)	86.03%	p < 0.001

Caption; K: Number of reports, n: Number of resistant isolates, N: Number of total isolates, LCI: 95% Lower, HCI: 95% Upper, P1: Heterogeneity p-value

**Table 2:** Evaluation of Publication Bias in Meta-Analysis.

Antibiotic	Egger Test	Begg test	Fail-Safe N	Trim & Fill (Proportion, 95%CI)	LFK index	LFK interpretation
Azithromycin	p = 0.019	p = 0.095	456	0.290 (0.195, 0.407)	2.336	major asymmetry
Clarithromycin	p = 0.649	p = 0.914	6345	0.305 (0.237, 0.382)	3.014	major asymmetry
Erythromycin	p = 0.062	p = 0.967	0	0.444 (0.322, 0.573)	2.882	major asymmetry

Caption; Egger/Begg: small-study effects; Fail-Safe N: robustness; Trim & Fill: adjusted proportion; LFK index/DOI plot: asymmetry (|LFK| < 1 none, 1–2 minor, > 2 major).

#### 3.3.2. Prevalence of clarithromycin resistance.

In the analysis of clarithromycin resistance, 76 reports (40 Studies) encompassing 3,923 *Mycobacterium* isolates were examined. Using a random-effects model, the estimated average resistance proportion was 0.305. The Q-test confirmed considerable heterogeneity among the outcomes (I² = 91.52%, p < 0.001). A forest plot illustrating the observed outcomes and random effects model estimates is presented in Fig 2The fill-and-trim method did not alter the estimated proportion of 0.305. Analysis of the studentized residuals showed no outliers, as none exceeded the threshold value of 3.407. Similarly, Cook’s distance analysis revealed that none of the studies significantly influenced the results. Tests for funnel plot asymmetry, including rank correlation and regression, did not indicate any bias (p = 0.914 and p = 0.649, respectively).

#### 3.3.3. Prevalence of erythromycin resistance.

Erythromycin resistance analysis included 752 isolates from 17 different reports (3 Studies). Using a random-effects model, the estimated average proportion of resistance was 0.503. The Q-test indicated substantial heterogeneity among outcomes (I² = 86.03%, p < 0.001). This variability is visually represented in the forest plot in Fig 2, which shows the observed outcomes and model estimates. When the fill-and-trim method was applied, the proportion was adjusted to 0.444 (95%CI, 0.322, 0.573). Analysis of the studentized residuals revealed no outliers, as none exceeded a value of 3.038. Additionally, Cook’s distance suggests that no individual study significantly influenced the results. Although the regression test indicated some asymmetry in the funnel plot (p = 0.967), this asymmetry was not confirmed by the rank correlation test (p = 0.062).

### 3.4. Subgroup analysis

This passage provides an in-depth summary of subgroup analyses related to antibiotic resistance (Fig 4). It explored the variations in resistance rates across different regions, the impact of various AST methods, temporal trends, and the influence of study quality on reported findings.

**Fig 3:**
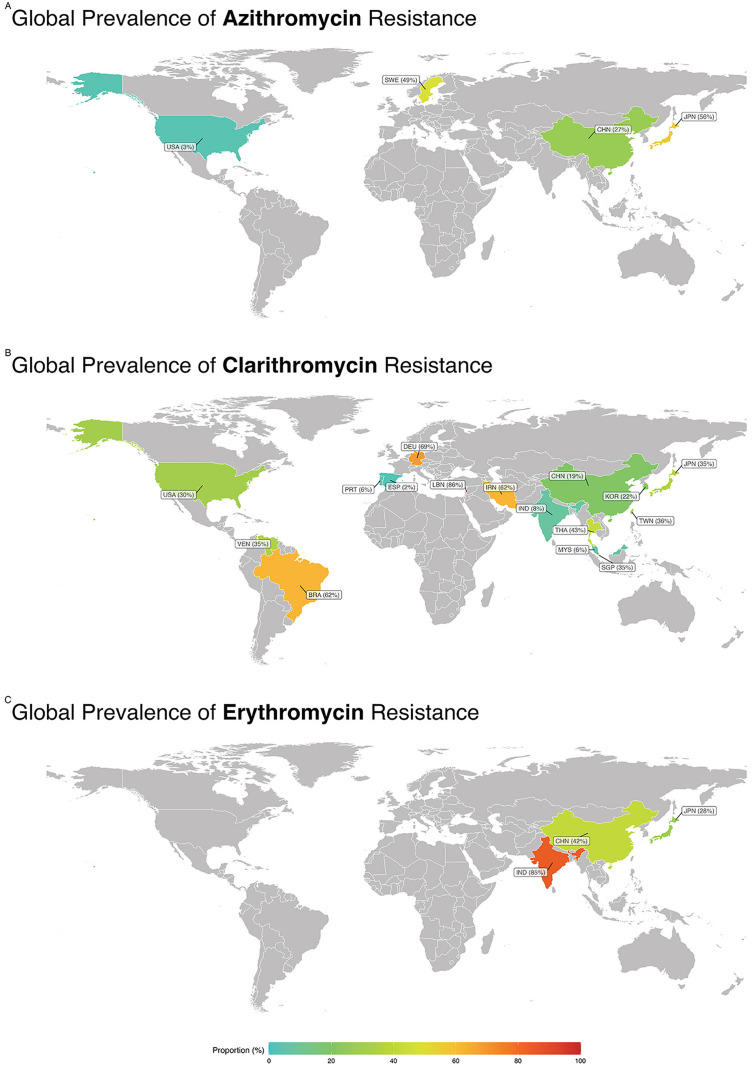
Global Prevalence of Macrolide Resistance in *Mycobacterium* Species. **A** show the prevalence of azithromycin resistance. The United States reported the lowest resistance (3.1%, light green), followed by China (27.5%, yellow-green), Sweden (40%, yellow), and Japan (56%, orange). **B** displays clarithromycin resistance across countries. The lowest resistance was observed in Spain (2%, light green), followed by Portugal (8%), the United States (8.5%), China (11%), and South Korea (22.5%) in varying shades of green to yellow-green. Moderate to high resistance was reported in Taiwan (30%), Singapore (35%), Germany (40%), Thailand (45%), Malaysia and Venezuela (60%), Brazil (62%), Iran (65%), India (80%, red), and Lebanon (85.8%, dark orange to red). **C** presents erythromycin resistance. Japan showed the lowest rate (27.5%, yellow-green), followed by China (41%, yellow), and India with the highest reported resistance at 84.6% (red). Only countries for which data were available are shown; countries in gray were not represented in the included studies. Color intensity reflects the proportion of resistance, ranging from low (green) to high (red). These figures represent national-level data, and within-country variations are not displayed. The global map was reprinted from OpenStreetMap data, provided under the Open Database License (ODbL), with permission from OpenStreetMap contributors, original copyright 2023, under the Creative Commons Attribution License (CC BY 4.0).

#### 3.4.1. Subgroup analysis based on countries.

Country-level patterns of macrolide resistance are illustrated in Fig 3. For azithromycin, two countries contributed data: Japan (1 study; 14/25 resistant; 56.0%) and China (16 studies; 142/690; 26.7%). Although the point estimates contrasted, the between-country test was not significant (P = 0.187), and heterogeneity was high within China (I² = 78.5%).

For clarithromycin, between-country differences were pronounced and statistically significant (P < 0.001). Very low resistance was observed in Spain (1/50; 2.0%) and Portugal (18/306; 5.9%), with similarly low rates in Malaysia (3/51; 5.9%) and India (2/26; 7.7%). Intermediate estimates were seen in South Korea (10 studies; 50/351; 15.6%), China (22 studies; 217/1,128; 19.4%), the USA (4 studies; 79/328; 40.6%; I² = 97.1%), Taiwan (2 studies; 18/41; 35.5%), and Singapore (2 studies; 117/348; 35.2%). High resistance was recorded in Germany (20/29; 69.0%), Brazil (4 studies; 56/90; 61.7%), Iran (8 studies; 160/308; 61.9%), and was extreme in Lebanon (23/24; 95.8%). Within-country heterogeneity was substantial for several settings (e.g., USA, Japan), whereas single-study countries necessarily showed I² = 0.

For erythromycin, country differences were also significant (P = 0.036). China (12 studies; 256/620) showed a pooled resistance of 41.7% (I² = 84.5%), Japan (3 studies; 72/106) 63.5% (I² = 82.0%), and India (2 studies; 22/26) the highest at 84.6% (I² = 0%).

#### 3.4.2. Subgroup Analysis Based on Continents.

**Fig 4:**
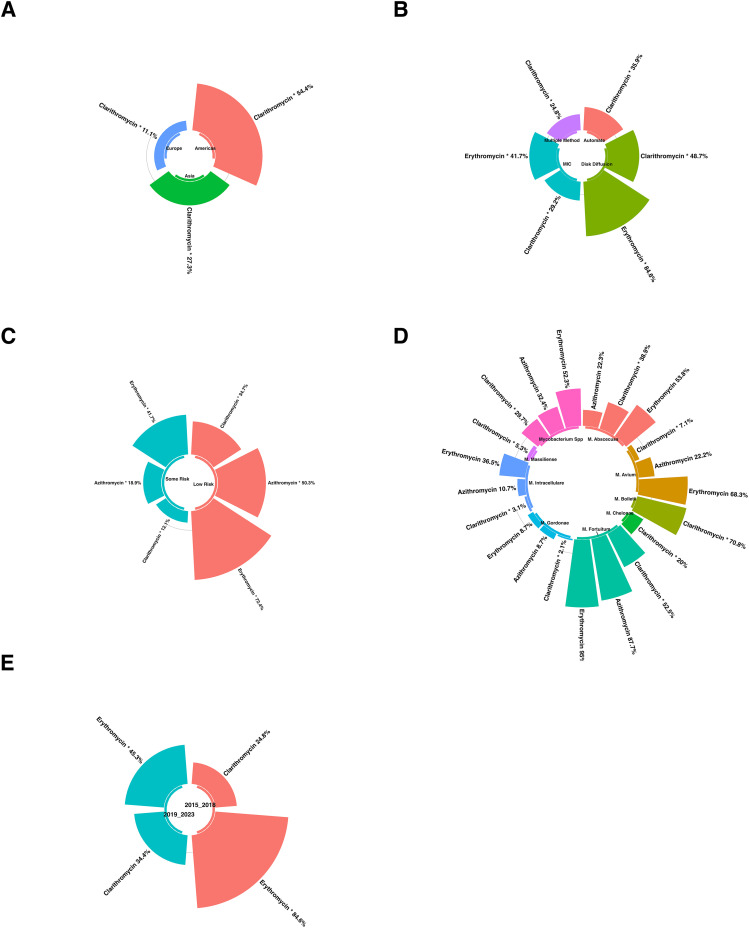
Subgroup analysis results were illustrated in figures; A: Compression of the prevalence of antibiotic-resistant Mycobacterium isolates between continents; B: Compression of the prevalence of antibiotic-resistant Mycobacterium isolates between AST methods; C: Compression of the prevalence of antibiotic-resistant Mycobacterium isolates between AST guidelines; D: Compression of the prevalence of antibiotic-resistant Mycobacterium isolates according to the risk of bias according to JBI; E: Compression of the prevalence of Mycobacterium based on species; F: Compression of the prevalence of Mycobacterium isolates before and after 2019.

Subgroup analysis revealed a statistically significant disparity in the prevalence of antibiotic resistance among various continents, including erythromycin. For erythromycin, the continent with the lowest resistance rate was NA, with a prevalence rate of 2.1%. Conversely, the continents with the highest resistance rates were observed in Asia, with a prevalence rate of 41.4% (Fig 4A).

#### 3.4.3. Subgroup Analysis Based on AST Method.

Subgroup analysis showed a statistically significant difference in erythromycin resistance rates across the various AST methods. The lowest resistance rate was reported in studies using MIC, with a prevalence of 41.7%. In contrast, the highest resistance rate was observed in studies using the disk diffusion method, with a prevalence of 84.6% (Fig 4B).

#### 3.4.5. Subgroup Analysis Based on Quality Group.

Subgroup analysis revealed a statistically significant disparity in the prevalence of antibiotic resistance among various quality groups, including azithromycin and clarithromycin. For the antibiotic azithromycin, the quality group with the lowest resistance rate was at risk, with a prevalence of 17.1%. Conversely, the low-risk group had the highest resistance rate, with a prevalence rate of 49.5%.

For clarithromycin, the quality group with the lowest resistance rate was at risk, with a prevalence of 14.1%. Conversely, the low-risk group had the highest resistance rate, with a prevalence rate of 35.7% (Fig. 4C).

#### 3.4.6. Subgroup analysis based on species.

Species-level subgroup analysis showed marked interspecies variation in macrolide resistance (azithromycin, clarithromycin, and erythromycin; Fig 4D). The species assessed were Mycobacterium spp. (non-speciated), *M. abscessus*, *M. avium*, *M. bolletii*, *M. chelonae*, *M. fortuitum*, *M. gordonae*, *M. intracellulare*, and *M. massiliense*. For azithromycin, resistance ranged from 8.7% in *M. gordonae* to 87.7% in *M. fortuitum*; intermediate proportions were 10.7% in *M. intracellulare*, 22.2% in M. avium, 22.3% in *M. abscessus*, and 32.4% in Mycobacterium spp. For clarithromycin, resistance spanned from 2.1% in *M. gordonae*, 3.1% in *M. intracellulare*, and 5.3% in *M. massiliense* to 70.8% in *M. bolletii*, with intermediate values of 7.1% in M. avium, 20% in *M. chelonae*, 29.7% in Mycobacterium spp., 38.9% in *M. abscessus*, and 52.5% in *M. fortuitum*. For erythromycin, resistance ranged from 8.7% in *M. gordonae* to 95% in *M. fortuitum*; additional observed proportions were 36.5% in *M. massiliense*, 52.3% in Mycobacterium spp., 53.8% in *M. abscessus*, and 68.3% in *M. avium*.

#### 3.4.7. Subgroup analysis based on year group.

The subgroup analysis revealed disparities in the prevalence of antibiotic resistance among various year groups. For azithromycin, the year group with the lowest rate of resistance was 2015–2018, exhibiting a prevalence rate of 18.9%, while conversely, the year group with the highest resistance rate was observed in 2019–2023, with a prevalence rate reaching 50.3%.

For clarithromycin, the year group with the lowest rate of resistance was 2015–2018, exhibiting a prevalence rate of 25.7%, while conversely, the year group with the highest resistance rate was observed in 2019–2023, with a prevalence rate reaching 42.5% (Fig 4E).

### 3.5. Meta-regression

**Fig 5:**
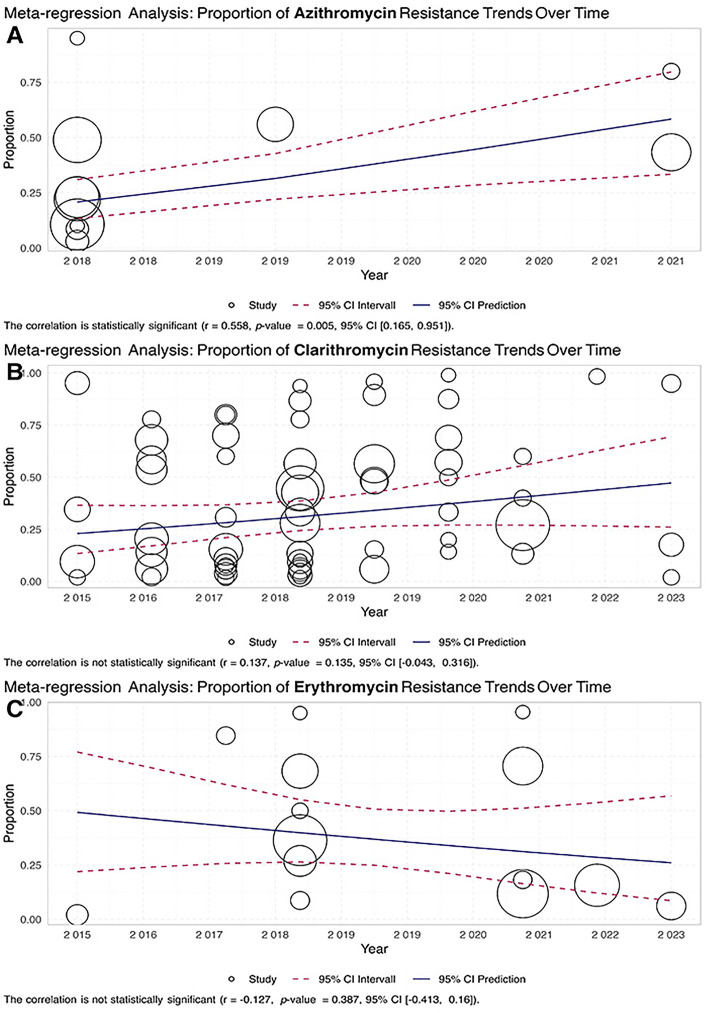
Meta-regression of macrolide resistance Mycobacterium isolates over the years; The results of the meta-regression analysis are presented in a scatter plot that shows the trend in the proportion of Mycobacterium-resistant isolates over the years: A: azithromycin, B: clarithromycin, C: erythromycin. Each point on the plot represents a specific study, with the x-axis indicating the year and the y-axis showing the proportion of resistant isolates. The scatter plot reveals how resistance rates have changed over time. A fitted regression line indicates the overall trend, highlighting whether resistance rates have increased, decreased, or remained stable.

A meta-regression assessed the association between calendar year and reported resistance proportions (Fig 5A–C). Only azithromycin showed a statistically significant positive trend over time (r = 0.558, p = 0.005, 95% CI: 0.165–0.951), indicating increasing resistance in more recent years (Fig 5A). No significant temporal trend was observed for clarithromycin (r = 0.137, p = 0.135, 95% CI: –0.043 to 0.316; Fig 5B) or erythromycin (r = –0.127, p = 0.387, 95% CI: –0.413 to 0.160; Fig 5C).

## 4. Discussion

The rising prevalence of macrolide resistance among *Mycobacterium* species has become a critical public health issue, particularly with the increasing number of infections caused by TB and NTM [25,42,61]. This systematic review and meta-analysis, which included 37 studies from 17 countries across three continents conducted between 2015 and 2023, provides a comprehensive assessment of macrolide resistance in *Mycobacterium* species. The analysis revealed considerable variability in resistance prevalence, influenced by geographic distribution, study period, AST methods, species, and study quality.

Our findings indicated significant resistance rates to macrolide antibiotics, including azithromycin (29%), clarithromycin (30.5%), and erythromycin (50.3%). The high heterogeneity observed (I² values of 80.98%, 91.52%, and 86.03%, respectively) highlights the complexity of resistance mechanisms and the influence of various factors such as geographic location, clinical settings, and study methodologies [62]. Regional disparities in antibiotic prescribing practices, differences in AST methods (e.g., MIC testing vs. disk diffusion), and variability in *Mycobacterium* species distribution contribute significantly to this heterogeneity. For example, subgroup analyses revealed resistance rates ranging from as low as 2–3% in some countries to over 80% in others, reflecting diverse local practices and resistance pressures.

Clinically, this heterogeneity argues against relying on pooled estimates for empirical therapy. Instead, clinicians should prioritize (i) species- and where relevant subspecies-level identification, (ii) locally generated AST results, and (iii) guideline-concordant methods that capture inducible macrolide resistance. From a public-health perspective, the large between-study variance supports reporting and interpreting subgroup-specific estimates (by region, species/subspecies, clinical setting, and AST methodology) and, where possible, emphasizing prediction intervals to convey the expected range in new settings. Finally, the observed heterogeneity highlights the need for strengthened regional surveillance and further standardization of AST procedures to improve comparability and guide context-appropriate treatment choices.

High resistance levels particularly to erythromycin illustrate a multifactorial challenge. The emergence of multidrug-resistant (MDR) strains suggests that resistance to one macrolide may be linked with cross-resistance to other antibiotic classes, complicating treatment approaches [63]. This concern is especially relevant to *M. abscessus*, which demonstrates intrinsic resistance to standard anti-tuberculous agents and alarmingly high resistance rates to key antibiotics such as clarithromycin and amikacin [64–66].

Additionally, bacterial efflux pumps, which actively expel antibiotics, have been identified as major contributors to resistance. Studies indicate that efflux pump inhibitors (EPIs) may restore susceptibility to certain antimycobacterial drugs, offering promising therapeutic adjuncts [67]. Studies have suggested that efflux pump inhibitors (EPIs) can enhance the effectiveness of existing antimycobacterial therapies, providing potential avenues for overcoming resistance. Therefore, localized surveillance and customized treatment strategies must address the regional variability in resistance levels [67]. Thus, the heterogeneity observed is not merely statistical but reflects underlying biological, epidemiological, and methodological diversity. These findings highlight the urgent need for localized resistance surveillance and species-specific treatment strategies tailored to regional resistance patterns.

The subgroup analyses in this review provided additional insights into the factors affecting macrolide resistance rates. Resistance patterns varied depending on the specific *Mycobacterium* species, geographic region, and AST methodology. This variability complicates data interpretation and underscores the need for standardized testing protocols to ensure consistent and reliable data [68,69]. Furthermore, significant variability in study quality, with some studies needing more rigorous methodologies, may introduce bias into the reported resistance rates [70].

The high intrinsic resistance of *Mycobacterium* species to commonly used macrolides such as azithromycin, clarithromycin, and erythromycin present a significant challenge in managing these infections. As macrolide resistance increases, it is imperative to implement ongoing surveillance and adjust the treatment guidelines accordingly [71]. Standardizing AST methodologies would enable more reliable comparisons across studies and improve the data quality used to inform treatment guidelines [25]. Incorporating molecular technology to identify resistant strains could also significantly enhance clinical outcomes by enabling timely adjustments to treatment regimens [72].

Significant associations were observed between resistance rates and various factors, including country, species, study quality, and year group. For example, azithromycin resistance is influenced by geographic and biological factors. Similarly, clarithromycin and erythromycin resistance have been linked to regional epidemiological conditions and AST methods. These findings emphasize the importance of considering multiple factors when evaluating resistance patterns to develop effective public health strategies and clinical practices to combat antibiotic resistance.

Regional disparities in macrolide resistance among *the Mycobacterium* species are particularly notable. For instance, clarithromycin resistance rates in the Spain are approximately 2%, whereas Lebanon reported rates as high as 96%. At the continental level, Europe had lower Clarithromycin resistance rates (11%), while Americas reported higher rates (54.4%). These findings underscore the need for region-specific healthcare and antibiotic policies to address the unique challenges posed by macrolide resistance in various settings.

Several studies have deepened our understanding of the regional differences in macrolide resistance among *Mycobacterium* species. For instance, Maurer et al. (2014) [73] identified significant methodological differences in susceptibility testing that could affect the reported resistance rates. Similarly, Ananta et al. (2018) [25] analyzed drug susceptibility patterns in *M. abscessus* isolates from Thailand and revealed high resistance levels consistent with regional trends. Pasipanodya et al. (2017) [74] conducted a systematic review examining the impact of geographic factors on treatment outcomes of *M. abscessus*, further illustrating how local factors influence resistance patterns. These studies highlight the importance of understanding local resistance dynamics to inform effective treatment strategies and public health interventions.

A comprehensive survey by Tu et al. (2022) [75] revealed significant variations in the resistance rates across different regions. Notably, parts of Asia have reported high resistance rates, reaching up to 74%, while resistance to first-line drugs in *Mycobacterium* species remains remarkably low in the United States. For example, Schwartz (2020) [76] reported resistance rates of less than 2% in the U.S. These findings align with the results of our study, underscoring the geographical disparities in resistance rates and reinforcing the critical need for tailored public health strategies to address these differences effectively.

Analysis of resistance rates among different *Mycobacterium* species revealed notable differences. For example, Clarithromycin resistance ranges from 2.1% in *M. gordonae* to 70.8% in *M. bolletii*. Azithromycin resistance was the lowest in *M. gordonae* (8.7%) and the highest in *M. fortuitum* (87.7%). Erythromycin resistance was minimal in *M. gordonae* (2.1%) but peaked at 95.0% in *M. fortuitum*. These species-specific differences highlight the need for targeted strategies to manage resistance effectively.

Across all included studies, *Mycobacterium spp*. were the most commonly reported group, with 38 studies from Asia, 6 from the Americas, 3 from Europe, and 3 from studies with unspecified regions. Regionally, Mycobacterium spp. were identified in 13 studies from China, 11 from Japan, 6 from South Korea, 4 from the United States, and one to two studies each from Thailand, Malaysia, Germany, Lebanon, Iran, India, and Spain. In contrast, subspecies were less frequently reported: *M. avium* was found in one study from Portugal (Europe), while *M. bolletii* and *M. massiliense* were each reported in one study with unspecified geographic origin. This geographic distribution demonstrates the dominance of *Mycobacterium* spp. in Asian studies, which may partly explain the higher macrolide resistance rates observed in that region.

These region-specific resistance trends may be further explained by the underlying genetic differences between prevalent species. For example, *M. abscessus* subsp. *abscessus*, which is more frequently isolated in East Asia, harbors a functional *erm* (41) gene that confers inducible resistance to macrolides such as clarithromycin. In contrast, *M. massiliense*, which lacks a functional *erm* (41) gene, is typically more susceptible to macrolides and has been identified in studies from the Americas. Additionally, high-level macrolide resistance may also result from point mutations in the 23S rRNA gene (*rrl*), which vary between species. These genetic distinctions provide a biologically plausible explanation for the observed geographic differences in resistance rates.

Methodological differences in AST levels can significantly affect the reported resistance rates. For example, the disk diffusion method showed an erythromycin resistance rate of 79.8% in isolates from Nigeria, underscoring the importance of standardized testing protocols to ensure reliable resistance data across studies and regions [77]. Genetic diversity within *Mycobacterium* species also contributes to variability in resistance patterns, further complicating the interpretation of susceptibility data [11,78]. Standardized AST protocols are essential to obtain reliable and comparable data on macrolide resistance. Variability in AST methods significantly impacts reported resistance rates, with discrepancies observed between processes, such as disk diffusion.

Recent studies have highlighted the influence of the COVID-19 pandemic on antibiotic resistance patterns, particularly to azithromycin. During the pandemic, resistance to azithromycin and clarithromycin has increased significantly, driven by widespread and often inappropriate use, misconceptions about their antiviral properties, and their use in managing secondary infections. This misuse has accelerated the development of resistant strains, underscoring the need for regulated antibiotic use during health crises to prevent further resistance [79–83]. The impact of the pandemic on resistance trends necessitates updated treatment guidelines and careful antibiotic stewardship during public health emergencies.

The finding that studies rated as low risk of bias reported higher macrolide resistance than those with some concerns is plausible and likely reflects methodological and contextual differences rather than a paradox. In our dataset, low-risk studies reported resistance of 49.5% for azithromycin and 35.7% for clarithromycin, versus 17.1% and 14.1% in studies at risk. Low-risk studies were more likely to (i) follow CLSI-concordant AST procedures (broth microdilution, with extended incubation to detect inducible macrolide resistance in the *M. abscessus* complex), (ii) achieve species/subspecies-level identification and, when available, molecular confirmation (e.g., *erm(41)*, *rrl* mutations), and (iii) provide complete reporting of non-susceptibility categories and denominators. Each of these practices increases detection of resistance that may be missed or under-reported in studies with methodological limitations (e.g., disk diffusion, short read times, genus-level identification). In addition, low-risk studies were more often clustered in regions and time periods with higher macrolide exposure (and thus higher resistance pressure) and were frequently conducted in tertiary referral centers that manage more refractory cases—both of which can elevate observed resistance independently of study quality. Together, these factors explain why stricter methods can yield higher resistance estimates and underscore the need to interpret pooled results in light of study quality, setting, and methods.

The variability in macrolide resistance across different years, regions, species, and methodological factors highlights the need for enhanced and continuous surveillance and integration of regional and global resistance data. Practical strategies to combat resistance include strengthening infection control in healthcare settings, standardizing global testing methods, developing localized treatment guidelines, and investing in new antimicrobial agents and alternative therapies [84]. Despite the strengths of this systematic review and meta-analysis, several important limitations should be acknowledged. First, the substantial heterogeneity observed across studies (I² > 80%) limits the generalizability of the pooled resistance estimates. This heterogeneity reflects diverse factors, including variability in study designs, patient populations, geographical settings, antibiotic prescribing practices, and AST methodologies. Although subgroup and meta-regression analyses were conducted to explore some sources of heterogeneity, a considerable proportion of unexplained variability remains. Second, the inclusion of only English-language publications may have introduced language bias, potentially excluding relevant studies published in other languages and limiting global representation. Third, the reliance on published literature raises the possibility of publication bias, as studies reporting high or significant resistance rates are more likely to be published. Although our assessments did not reveal major funnel plot asymmetry, the influence of unpublished negative findings cannot be entirely excluded. Fourth, Species-specific pooling was constrained by small study numbers. To avoid unstable heterogeneity estimates, we required ≥3 studies per species; after excluding species with one study, three species-level subgroups remained, and after excluding those with one or two studies, only one subgroup met criteria. For species with <3 studies, we did not pool results and instead provide descriptive summaries so that findings for all species remain visible.

Fourth, species-level data were not consistently reported across studies, which constrained our ability to conduct in-depth, species-specific resistance analyses and evaluate the full spectrum of resistance patterns across clinically relevant Mycobacterium subspecies. Fifth, there was notable variation in AST methodologies ranging from MIC testing and disk diffusion to automated systems with some studies lacking methodological detail. These differences can significantly influence reported resistance rates and complicate direct study comparisons.

Finally, and importantly, the analysis did not include clinical treatment outcomes such as cure rates, treatment failure, relapse, or mortality. Most included studies focused solely on microbiological resistance profiles without linking them to patient-level therapeutic outcomes. As a result, it was impossible to assess how in vitro resistance translated into real-world treatment success or failure. This represents a critical gap, as resistance patterns must ultimately be interpreted in the context of clinical efficacy. Future research should integrate antimicrobial susceptibility data with standardized treatment outcomes to guide evidence-based therapy and improve patient care.

Together, these limitations highlight the need for standardized global AST protocols, more rigorous and transparent study reporting, the inclusion of clinical endpoints, and the design of prospective multicenter studies that capture both microbiological and clinical data. Addressing these issues will improve the comparability of resistance estimates and ensure that surveillance efforts effectively inform clinical decision-making and public health strategies.

## 5. Conclusion

This systematic review and meta-analysis of 37 studies across 17 countries revealed substantial macrolide resistance among *Mycobacterium* species, with pooled rates of 29.0% for azithromycin, 30.5% for clarithromycin, and 50.3% for erythromycin. Resistance patterns varied widely by species, region, study quality, and AST methodology, with prevalence ranging from <5% in some countries to >80% in others. The high heterogeneity (I² > 80%) reflects underlying biological, methodological, and epidemiological diversity and limits the generalizability of pooled estimates.

Translationally, these findings highlight the urgent need for strengthened laboratory capacity, routine and standardized AST at the species/subspecies level, and region-specific treatment guidelines that reflect local resistance dynamics. Continuous surveillance and methodological standardization are essential to generate reliable, comparable data, while integration of clinical outcomes and molecular diagnostics in future prospective studies will be critical to guide effective therapy. Coordinated local, regional, and global actions are needed to contain macrolide resistance and improve management of *Mycobacterium* species infections.

## Supporting information

S1 File Supplementary Data. Additional supplementary material, including extracted datasets, subgroup analyses, and extended methodological details.
(DOCX)

S2 File List of Studies Included in the Systematic Review and Meta-analysis. Detailed information on all studies included, including first author, year, country, study design, sample size, and macrolide resistance data.
(CSV)

S3 File PRISMA Checklist. The Preferred Reporting Items for Systematic Reviews and Meta-Analyses (PRISMA) checklist was completed for this study.
(DOCX)
