# Supplementary material for: Global trends in the proportion of macrolide-resistant Mycobacterium Species: A systematic review and meta-analysis
Source: PLoS One. 2025 Nov 7;20(11):e0333521. doi: 10.1371/journal.pone.0333521 (PMC12594324; doi:10.1371/journal.pone.0333521)
Supplement: S1 File — Additional supplementary material, including extracted datasets, subgroup analyses, and extended methodological details. (DOCX) [file pone.0333521.s001.docx]

**1. Search syntax was used for the literature review in each online database.**

("*Mycobacterium**" OR "*M. bovis*" OR "*M. tuberculosis*" OR "*M. leprae*" OR "*M. avium*" OR "*M. intracellulare*" OR "*M. abscessus*" OR *"M. Chelonae"* OR "*M. fortuitum*" OR "*M. kansasii*" OR "*M. marinum*" OR "*M. ulcerans*" OR "*M. smegmatis*" OR "*M. xenopi*" OR "*M. gordonae*" OR "*M. haemophilum*" OR "*M. terrae*" OR "*M. africanum*" OR "*M. asiaticum*" OR "*M. malmoense*" OR "*M. szulgai*" OR "*M. simiae*" OR "*M. celatum*" OR "*M. shimoidei*" OR "*M. xenopi*" OR "*M. lentiflavum*" OR "*M. arupense*") AND (macrolide* OR azithromycin OR clarithromycin OR erythromycin OR roxithromycin OR telithromycin OR spiramycin OR fidaxomicin) AND (resistan* OR susceptib*)

**2. Comprehensive Antibiotic-Specific Meta-analysis Results**

### 2.1 Prevalence of Azithromycin Resistance

A total of 715 isolates from 17 studies were included in the analysis of azithromycin resistance. The pooled prevalence, estimated using a random-effects model, was 0.290 (95% CI: 0.195–0.407), and the average outcome differed significantly from zero (z = –3.366, p < 0.001). The Q-test indicated significant heterogeneity among studies (Q(16) = 84.113, I² = 80.98%, p < 0.001). A forest plot of the study-specific and pooled estimates is presented in Figure 2. Application of the trim-and-fill method yielded no change in the pooled prevalence (0.290; 95% CI: 0.195–0.407). Studentized residuals showed no outliers (all < 2.974), and Cook’s distance values indicated that no single study exerted undue influence. Funnel plot asymmetry was suggested by the regression test (p = 0.019) but not confirmed by the rank correlation test (p = 0.095).

### Prevalence of Clarithromycin Resistance

The analysis of clarithromycin resistance included 3,923 isolates from 76 studies. The pooled prevalence was 0.305 (95% CI: 0.237–0.382), significantly different from zero (z = –4.690, p < 0.001). Substantial heterogeneity was detected (Q(75) = 884.878, I² = 91.52%, p < 0.001). The forest plot of study-level and pooled results is shown in Figure 2. The trim-and-fill method produced no adjustment (0.305; 95% CI: 0.237–0.382). No outliers were detected (studentized residuals < 3.407), and Cook’s distances indicated no overly influential studies. Both the regression test (p = 0.649) and the rank correlation test (p = 0.914) suggested no funnel plot asymmetry.

### Prevalence of Erythromycin Resistance

For erythromycin, 752 isolates from 17 studies were analyzed. The pooled prevalence was 0.503 (95% CI: 0.375–0.629), and the average outcome did not differ significantly from zero (z = 0.039, p = 0.969). Considerable heterogeneity was observed (Q(16) = 114.520, I² = 86.03%, p < 0.001). The forest plot is presented in Figure 2. After applying the trim-and-fill method, the adjusted prevalence was 0.444 (95% CI: 0.322–0.573). No outliers were identified (studentized residuals < 2.974), and Cook’s distances confirmed that no study was overly influential. Funnel plot asymmetry was not indicated by either the regression test (p = 0.967) or the rank correlation test (p = 0.062).

**Supplementary Table 1:** Characteristics and Extracted Data of Studies Included in the Meta-analysis

| Author | Year | Countries | AST method | AST Guideline | Quality Group | Azithromycin | Clarithromycin | Erythromycin | Reference |
| --- | --- | --- | --- | --- | --- | --- | --- | --- | --- |
| B. A. Brown-Elliott, et al. | 2015 | United States | Multiple Method | CLSI | Low Risk | NA | 15 | NA | (1) |
| M. Schwartz, et al. | 2018 | United States | Multiple Method | CLSI | Low Risk | NA | 26 | NA | (2) |
| H. Y. Kim, et al. | 2016 | South Korea | Multiple Method | CLSI | Low Risk | NA | 22 | NA | (3) |
| S. Daniel-Wayman, et al. | 2019 | United States | MIC | CLSI | Low Risk | NA | 34 | NA | (4) |
| P. Ananta, et al. | 2018 | Thailand | Multiple Method | CLSI | Low Risk | NA | 29 | NA | (5) |
| S. D. Jayasingam, et al. | 2017 | Malaysia | MIC | CLSI | Low Risk | NA | 3 | NA | (6) |
| K. I. Uchiya, et al. | 2018 | Japan | Automate | CLSI | Low Risk | NA | 4 | NA | (7) |
| G. Li, et al. | 2017 | China | Multiple Method | CLSI | Low Risk | NA | 15 | NA | (8) |
| N. Wetzstein, et al. | 2020 | Germany | Multiple Method | CLSI | Low Risk | NA | 20 | NA | (9) |
| S. Yoshida, et al. | 2019 | Japan | MIC | CLSI | Low Risk | 14 | 12 | NA | (10) |
| G. Wei, et al. | 2015 | China | Multiple Method | CLSI | Low Risk | NA | 1 | NA | (11) |
| A. Aono, et al. | 2019 | Japan | Multiple Method | CLSI | Low Risk | NA | 48 | NA | (12) |
| S. Hatakeyama, et al. | 2017 | Japan | Multiple Method | CLSI | Low Risk | NA | 4 | NA | (13) |
| S. Y. Kim, et al. | 2016 | South Korea | Multiple Method | CLSI | Low Risk | NA | 2 | NA | (13) |
| L. Luo, et al. | 2016 | China | Automate | CLSI | Low Risk | NA | 36 | NA | (14) |
| Q. Guo, et al. | 2018 | China | Multiple Method | CLSI | Low Risk | NA | 28 | NA | (15) |
| Y. J. Guo, et al. | 2022 | China | MIC | CLSI | Low Risk | NA | 59 | NA | (16) |
| L. C. Chen, et al. | 2020 | Taiwan | Automate | NA | Low Risk | NA | 16 | NA | (17) |
| B. A. Brown-Elliott, et al. | 2017 | United States | MIC | CLSI | Low Risk | NA | 4 | NA | (18) |
| S. Yoshida, et al. | 2015 | Japan | NA | CLSI | Low Risk | NA | 99 | NA | (19) |
| Y. Li, et al. | 2016 | China | Multiple Method | CLSI | Low Risk | NA | 16 | NA | (20) |
| K. Kamada, et al. | 2021 | Japan | NA | CLSI | Low Risk | NA | NA | 60 | (21) |
| K. Kamada, et al. | 2021 | Japan | NA | CLSI | Low Risk | NA | NA | 2 | (21) |
| K. Kamada, et al. | 2021 | Japan | NA | CLSI | Low Risk | NA | NA | 10 | (21) |
| G. F. Araj, et al. | 2019 | Lebanon | Multiple Method | CLSI | Low Risk | NA | 23 | NA | (22) |
| T. Hirama, et al. | 2016 | Japan | Multiple Method | CLSI | Low Risk | NA | 12 | NA | (23) |
| R. Ghosh, et al. | 2017 | India | Disk Diffusion | NA | Low Risk | NA | 1 | 11 | (24) |
| NA | 2019 | Portugal | Multiple Method | CLSI | Low Risk | NA | 9 | NA | (25) |
| K. L. Chew, et al. | 2017 | NA | MIC | CLSI | Low Risk | NA | 16 | NA | (26) |
| K. L. Chew, et al. | 2017 | NA | MIC | CLSI | Low Risk | NA | 3 | NA | (26) |
| K. L. Chew, et al. | 2017 | NA | MIC | CLSI | Low Risk | NA | 1 | NA | (26) |
| F. Li, et al. | 2018 | China | MIC | CLSI | Some Risk | 12 | 7 | 14 | (27) |
| F. Li, et al. | 2018 | China | MIC | CLSI | Some Risk | 0 | 0 | 2 | (27) |
| F. Li, et al. | 2018 | China | MIC | CLSI | Some Risk | 9 | 7 | 9 | (27) |
| F. Li, et al. | 2018 | China | MIC | CLSI | Some Risk | 14 | 6 | 43 | (27) |
| F. Li, et al. | 2018 | China | MIC | CLSI | Some Risk | 17 | 5 | 58 | (27) |
| F. Li, et al. | 2018 | China | MIC | CLSI | Some Risk | 2 | 0 | 2 | (27) |
| A. D. Khosravi, et al. | 2018 | Iran | Disk Diffusion | CLSI | Low Risk | NA | 26 | NA | (28) |
| A. D. Khosravi, et al. | 2018 | Iran | Disk Diffusion | CLSI | Low Risk | NA | 7 | NA | (28) |
| Q. Lei, et al. | 2021 | China | MIC | NA | Low Risk | 13 | 4 | NA | (29) |
| Q. Lei, et al. | 2021 | China | MIC | NA | Low Risk | 4 | 2 | NA | (29) |
| E. Shipitsyna, et al. | 2020 | South Korea | MIC | CLSI | Low Risk | NA | 3 | NA | (30) |
| E. Shipitsyna, et al. | 2020 | South Korea | MIC | CLSI | Low Risk | NA | 1 | NA | (30) |
| J. Park, et al. | 2017 | South Korea | MIC | CLSI | Low Risk | NA | 4 | NA | (31) |
| J. Park, et al. | 2017 | South Korea | MIC | CLSI | Low Risk | NA | 5 | NA | (31) |
| N. F. Garcia De Carvalho, et al. | 2016 | Brazil | MIC | CLSI | Low Risk | NA | 21 | NA | (32) |
| N. F. Garcia De Carvalho, et al. | 2016 | Brazil | MIC | CLSI | Low Risk | NA | 7 | NA | (32) |
| T. Asakura, et al. | 2019 | Japan | Multiple Method | CLSI | Low Risk | NA | 15 | NA | (33) |
| Alba Ruedas-López et al, | 2023 | Spain | Multiple Method | CLSI | Low Risk | NA | 1 | NA | (34) |
| J. Watanabe, et al. | 2023 | Japan | Multiple Method | CLSI | Low Risk | NA | 6 | NA | (35) |
| R. Ghosh, et al. | 2017 | India | Disk Diffusion | NA | Low Risk | NA | 1 | 11 | (24) |
| V Durão et al. | 2019 | Portugal | Multiple Method | CLSI | Low Risk | NA | 9 | NA | (25) |
| K. L. Chew, et al. | 2017 | NA | MIC | CLSI | Low Risk | NA | 16 | NA | (26) |
| K. L. Chew, et al. | 2017 | NA | MIC | CLSI | Low Risk | NA | 3 | NA | (26) |
| K. L. Chew, et al. | 2017 | NA | MIC | CLSI | Low Risk | NA | 1 | NA | (26) |
| A. Cheng, et al. | 2019 | Taiwan | MIC | CLSI | Low Risk | NA | 2 | NA | (36) |
| K. Fujiwara, et al. | 2023 | NA | MIC | NA | Low Risk | NA | 38 | NA | (37) |

**Supplementary Table 2:** Prevalence of Antibiotic Resistance

| Category | Subgroup | K (n, N) | Proportion 95%CI(LCI, HCI) | I² | P1 | P2 |
| --- | --- | --- | --- | --- | --- | --- |
|  |  | Azithromycin |  |  |  |  |
| Overall | NA | 17 (156, 715) | 0.290 (0.195, 0.407) | 80.98% | p<0.001 | NA |
| Country | Japan | 1 (14, 25) | 0.560 (0.366, 0.737) | 0.00% | p>0.999 | p=0.187 |
|  | China | 16 (142, 690) | 0.267 (0.179, 0.379) | 78.50% | p<0.001 |  |
| Species | *mycobacterium spp* | 10 (98, 400) | 0.324 (0.192, 0.492) | 83.15% | p<0.001 | p=0.053 |
|  | *m. abscecuss* | 2 (12, 56) | 0.223 (0.132, 0.350) | 0.00% | p=0.515 |  |
|  | *m. fortuitum* | 2 (13, 14) | 0.877 (0.557, 0.976) | 0.00% | p=0.395 |  |
|  | *m. avium* | 1 (14, 63) | 0.222 (0.136, 0.341) | 0.00% | p>0.999 |  |
|  | *m. intracellulare* | 1 (17, 159) | 0.107 (0.068, 0.165) | 0.00% | p>0.999 |  |
|  | *m. gordonae* | 1 (2, 23) | 0.087 (0.022, 0.289) | 0.00% | p>0.999 |  |
| Year Group | 2019_2023 | 5 (48, 95) | 0.503 (0.391, 0.614) | 10.93% | p=0.344 | p<0.001 |
|  | 2015_2018 | 12 (108, 620) | 0.189 (0.125, 0.274) | 69.29% | p<0.001 |  |
|  |  | Clarithromycin |  |  |  |  |
| Overall | NA | 76 (1071, 3923) | 0.305 (0.237, 0.382) | 91.52% | p<0.001 | NA |
| Country | United States | 4 (79, 328) | 0.406 (0.051, 0.897) | 97.09% | p<0.001 | p<0.001 |
|  | South Korea | 10 (50, 351) | 0.156 (0.073, 0.301) | 81.92% | p<0.001 |  |
|  | Thailand | 1 (29, 68) | 0.426 (0.315, 0.546) | 0.00% | p>0.999 |  |
|  | Malaysia | 1 (3, 51) | 0.059 (0.019, 0.167) | 0.00% | p>0.999 |  |
|  | Japan | 8 (200, 567) | 0.353 (0.138, 0.650) | 95.53% | p<0.001 |  |
|  | China | 22 (217, 1128) | 0.194 (0.115, 0.307) | 88.12% | p<0.001 |  |
|  | Germany | 1 (20, 29) | 0.690 (0.503, 0.830) | 0.00% | p>0.999 |  |
|  | Taiwan | 2 (18, 41) | 0.355 (0.073, 0.793) | 81.44% | p=0.020 |  |
|  | Lebanon | 1 (23, 24) | 0.958 (0.756, 0.994) | 0.00% | p>0.999 |  |
|  | India | 2 (2, 26) | 0.077 (0.019, 0.261) | 0.00% | p>0.999 |  |
|  | Portugal | 2 (18, 306) | 0.059 (0.037, 0.091) | 0.00% | p>0.999 |  |
|  | Iran | 8 (160, 308) | 0.619 (0.389, 0.806) | 88.25% | p<0.001 |  |
|  | Brazil | 4 (56, 90) | 0.617 (0.511, 0.712) | 0.00% | p=0.528 |  |
|  | Spain | 1 (1, 50) | 0.020 (0.003, 0.129) | 0.00% | p>0.999 |  |
|  | Singapore | 2 (117, 348) | 0.352 (0.203, 0.538) | 90.95% | p<0.001 |  |
| Continent | Americas | 8 (135, 418) | 0.544 (0.236, 0.822) | 94.84% | p<0.001 | p=0.033 |
|  | Asia | 57 (819, 2912) | 0.273 (0.208, 0.351) | 90.17% | p<0.001 |  |
|  | Europe | 4 (39, 385) | 0.111 (0.018, 0.463) | 95.16% | p<0.001 |  |
| AST Method | Multiple Method | 20 (359, 1614) | 0.248 (0.156, 0.371) | 93.56% | p<0.001 | p=0.511 |
|  | Mic | 46 (489, 1916) | 0.292 (0.205, 0.397) | 89.17% | p<0.001 |  |
|  | Automate | 3 (56, 157) | 0.359 (0.076, 0.794) | 94.83% | p<0.001 |  |
|  | Disk Diffusion | 6 (68, 132) | 0.487 (0.253, 0.727) | 74.01% | p=0.002 |  |
| Species | *mycobacterium spp* | 48 (689, 2548) | 0.297 (0.210, 0.401) | 92.44% | p<0.001 | p=0.019 |
|  | *m. abscecuss* | 11 (213, 582) | 0.389 (0.234, 0.569) | 88.75% | p<0.001 |  |
|  | *m. avium* | 2 (15, 216) | 0.071 (0.043, 0.115) | 0.00% | p=0.343 |  |
|  | *m. bolletii* | 2 (10, 14) | 0.708 (0.426, 0.887) | 0.00% | p=0.486 |  |
|  | *m. massiliense* | 2 (6, 105) | 0.053 (0.009, 0.268) | 67.56% | p=0.079 |  |
|  | *m. fortuitum* | 8 (132, 271) | 0.525 (0.305, 0.736) | 86.63% | p<0.001 |  |
|  | *m. intracellulare* | 1 (5, 159) | 0.031 (0.013, 0.073) | 0.00% | p>0.999 |  |
|  | *m. gordonae* | 1 (0, 23) | 0.021 (0.001, 0.259) | 0.00% | p>0.999 |  |
|  | *m. chelonae* | 1 (1, 5) | 0.200 (0.027, 0.691) | 0.00% | p>0.999 |  |
| Year Group | 2015_2018 | 52 (652, 2796) | 0.257 (0.185, 0.346) | 91.88% | p<0.001 | p=0.048 |
|  | 2019_2023 | 24 (419, 1127) | 0.425 (0.285, 0.579) | 90.85% | p<0.001 |  |
|  |  | Erythromycin |  |  |  |  |
| Overall | NA | 17 (350, 752) | 0.503 (0.375, 0.629) | 86.03% | p<0.001 | NA |
| Country | Japan | 3 (72, 106) | 0.635 (0.193, 0.927) | 81.98% | p=0.004 | p=0.036 |
|  | India | 2 (22, 26) | 0.846 (0.655, 0.941) | 0.00% | p>0.999 |  |
|  | China | 12 (256, 620) | 0.417 (0.293, 0.552) | 84.47% | p<0.001 |  |
| AST Method | Disk Diffusion | 2 (22, 26) | 0.846 (0.655, 0.941) | 0.00% | p>0.999 | p=0.012 |
|  | Mic | 12 (256, 620) | 0.417 (0.293, 0.552) | 84.47% | p<0.001 |  |
| Species | *mycobacterium spp* | 10 (211, 429) | 0.523 (0.342, 0.698) | 87.13% | p<0.001 | p=0.213 |
|  | *m. abscecuss* | 3 (27, 69) | 0.538 (0.163, 0.874) | 81.75% | p=0.004 |  |
|  | *m. fortuitum* | 1 (9, 9) | 0.950 (0.525, 0.997) | 0.00% | p>0.999 |  |
|  | *m. avium* | 1 (43, 63) | 0.683 (0.558, 0.785) | 0.00% | p>0.999 |  |
|  | *m. intracellulare* | 1 (58, 159) | 0.365 (0.294, 0.442) | 0.00% | p>0.999 |  |
|  | *m. gordonae* | 1 (2, 23) | 0.087 (0.022, 0.289) | 0.00% | p>0.999 |  |
| Year Group | 2019_2023 | 3 (72, 106) | 0.635 (0.193, 0.927) | 81.98% | p=0.004 | p=0.456 |
|  | 2015_2018 | 14 (278, 646) | 0.478 (0.348, 0.611) | 84.69% | p<0.001 |  |
| Caption; K: *Number of reports*, n: *Number of resistant isolates*, N: *Number of total isolates*, LCI/HCI: *95% CI*, P1: *P-value vs zero*, P1: *heterogeneity*, P2: *between-group test*. | | | | | | |


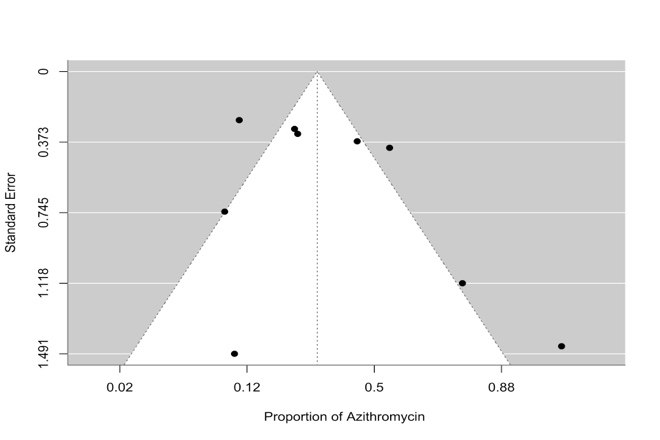

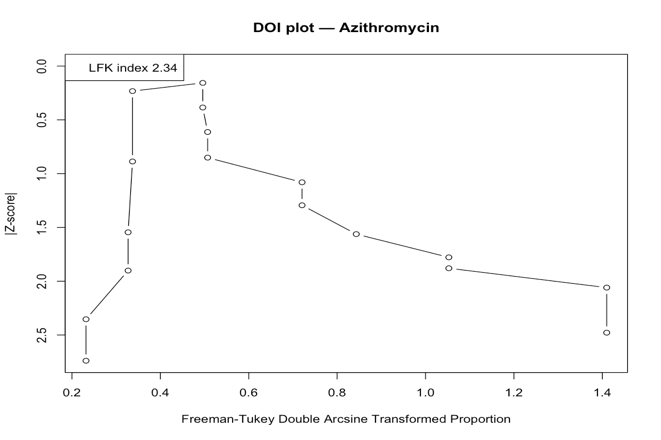

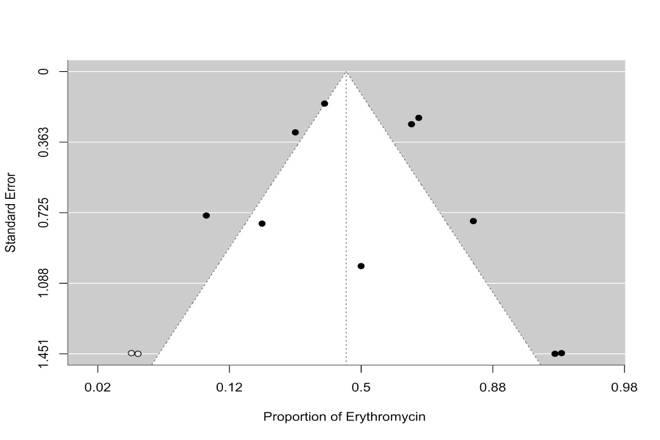

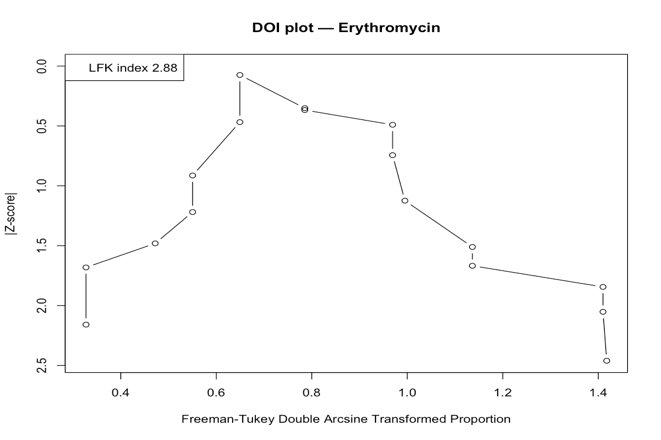

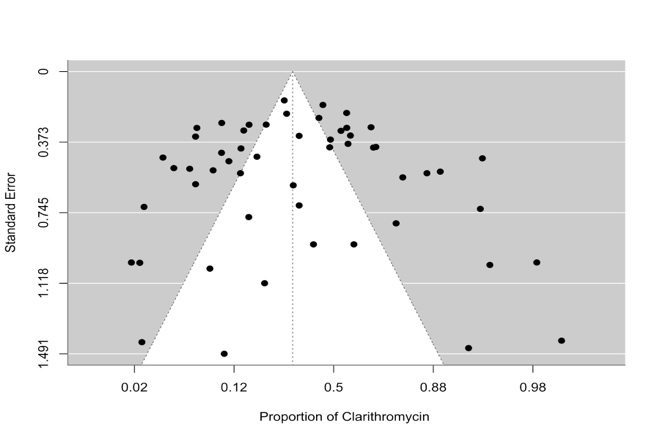

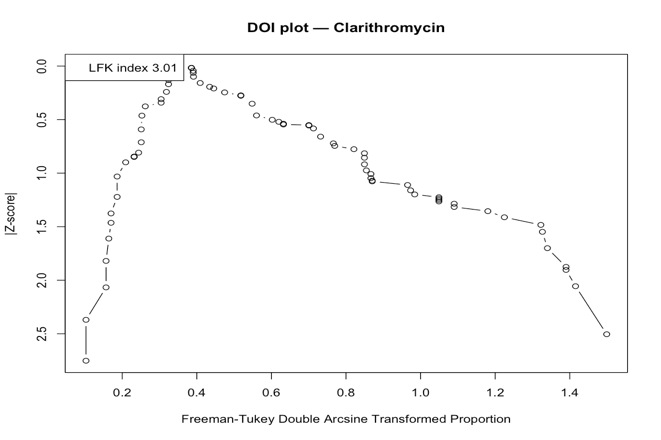


**Supplementary Figure 1:** Funnel plots for investigation of publication bias

1. Brown-Elliott BA, Hanson K, Vasireddy S, Iakhiaeva E, Nash KA, Vasireddy R, et al. Absence of a functional erm gene in isolates of Mycobacterium immunogenum and the Mycobacterium mucogenicum group, based on in vitro clarithromycin susceptibility. Journal of Clinical Microbiology. 2015;53(3):875-8.

2. Schwartz M, Fisher S, Story-Roller E, Lamichhane G, Parrish N. Activities of dual combinations of antibiotics against multidrug-resistant nontuberculous mycobacteria recovered from patients with cystic fibrosis. Microbial Drug Resistance. 2018;24(8):1191-7.

3. Kim H, Lee S, Kim B, Kook Y. Allele-specific duplex polymerase chain reaction to differentiate Mycobacterium abscessus subspecies and to detect highly clarithromycin-resistant isolates. Indian Journal of Medical Microbiology. 2016;34(3):369-74.

4. Daniel-Wayman S, Shallom S, Azeem N, Olivier KN, Zelazny AM, Prevots DR. Amikacin exposure and susceptibility of macrolide-resistant Mycobacterium abscessus. ERJ Open Research. 2019;5(2).

5. Ananta P, Kham-Ngam I, Chetchotisakd P, Chaimanee P, Reechaipichitkul W, Namwat W, et al. Analysis of drug-susceptibility patterns and gene sequences associated with clarithromycin and amikacin resistance in serial Mycobacterium abscessus isolates from clinical specimens from Northeast Thailand. PLoS One. 2018;13(11):e0208053.

6. Jayasingam SD, Zin T, Ngeow YF. Antibiotic resistance in Mycobacterium Abscessus and Mycobacterium Fortuitum isolates from Malaysian patients. The International Journal of Mycobacteriology. 2017;6(4):387-90.

7. Uchiya K-i, Asahi S, Futamura K, Hamaura H, Nakagawa T, Nikai T, et al. Antibiotic susceptibility and genotyping of Mycobacterium avium strains that cause pulmonary and disseminated infection. Antimicrobial Agents and Chemotherapy. 2018;62(4):10.1128/aac. 02035-17.

8. Li G, Pang H, Guo Q, Huang M, Tan Y, Li C, et al. Antimicrobial susceptibility and MIC distribution of 41 drugs against clinical isolates from China and reference strains of nontuberculous mycobacteria. International journal of antimicrobial agents. 2017;49(3):364-74.

9. Wetzstein N, Kohl TA, Schultze TG, Andres S, Bellinghausen C, Hügel C, et al. Antimicrobial susceptibility and phylogenetic relations in a German cohort infected with Mycobacterium abscessus. Journal of Clinical Microbiology. 2020;58(12):10.1128/jcm. 01813-20.

10. Yoshida S, Tsuyuguchi K, Chikamatsu K, Aono A, Takaki A, Mitarai S, et al. Antimicrobial susceptibility patterns and MICs among non-photochromogenic rapidly growing Mycobacteroides and Mycolicibacterium species. Journal of Medical Microbiology. 2019;68(9):1279-86.

11. Wei G, Huang M, Wang G, Huo F, Dong L, Li Y, et al. Antimicrobial susceptibility testing and genotyping of Mycobacterium avium isolates of two tertiary tuberculosis designated hospital, China. Infection, Genetics and Evolution. 2015;36:141-6.

12. Aono A, Morimoto K, Chikamatsu K, Yamada H, Igarashi Y, Murase Y, et al. Antimicrobial susceptibility testing of Mycobacteroides (Mycobacterium) abscessus complex, Mycolicibacterium (Mycobacterium) fortuitum, and Mycobacteroides (Mycobacterium) chelonae. Journal of Infection and Chemotherapy. 2019;25(2):117-23.

13. Hatakeyama S, Ohama Y, Okazaki M, Nukui Y, Moriya K. Antimicrobial susceptibility testing of rapidly growing mycobacteria isolated in Japan. BMC infectious diseases. 2017;17:1-7.

14. Luo L, Li B, Chu H, Huang D, Zhang Z, Zhang J, et al. Characterization of Mycobacterium abscessus subtypes in Shanghai of China: drug sensitivity and bacterial epidemicity as well as clinical manifestations. Medicine. 2016;95(3):e2338.

15. Guo Q, Chu H, Ye M, Zhang Z, Li B, Yang S, et al. The clarithromycin susceptibility genotype affects the treatment outcome of patients with Mycobacterium abscessus lung disease. Antimicrobial Agents and Chemotherapy. 2018;62(5):10.1128/aac. 02360-17.

16. Guo Y, Cao Y, Liu H, Yang J, Wang W, Wang B, et al. Clinical and microbiological characteristics of Mycobacterium kansasii pulmonary infections in China. Microbiology Spectrum. 2022;10(1):e01475-21.

17. Chen L-C, Huang H-N, Yu C-J, Chien J-Y, Hsueh P-R. Clinical features and treatment outcomes of Mycobacterium chimaera lung disease and antimicrobial susceptibility of the mycobacterial isolates. Journal of Infection. 2020;80(4):437-43.

18. Brown-Elliott BA, Philley JV, Griffith DE, Thakkar F, Wallace Jr RJ. In vitro susceptibility testing of bedaquiline against Mycobacterium avium complex. Antimicrobial agents and chemotherapy. 2017;61(2):10.1128/aac. 01798-16.

19. Brown-Elliott BA, Vasireddy S, Vasireddy R, Iakhiaeva E, Howard ST, Nash K, et al. Utility of sequencing the erm(41) gene in isolates of Mycobacterium abscessus subsp. abscessus with low and intermediate clarithromycin MICs. J Clin Microbiol. 2015;53(4):1211-5.

20. Li Y, Pang Y, Tong X, Zheng H, Zhao Y, Wang C. Mycobacterium kansasii subtype I is associated with clarithromycin resistance in China. Frontiers in microbiology. 2016;7:2097.

21. Kamada K, Yoshida A, Iguchi S, Arai Y, Uzawa Y, Konno S, et al. Nationwide surveillance of antimicrobial susceptibility of 509 rapidly growing mycobacteria strains isolated from clinical specimens in Japan. Scientific reports. 2021;11(1):12208.

22. Araj GF, Baba OZ, Itani LY, Avedissian AZ, Sobh GM. Non-tuberculous mycobacteria profiles and their anti-mycobacterial resistance at a major medical center in Lebanon. The Journal of Infection in Developing Countries. 2019;13(07):612-8.

23. Hirama T, Shiono A, Egashira H, Kishi E, Hagiwara K, Nakamura H, et al. PCR-based rapid identification system using bridged nucleic acids for detection of clarithromycin-resistant Mycobacterium avium-M. intracellulare complex isolates. Journal of Clinical Microbiology. 2016;54(3):699-704.

24. Ghosh R, Das S, De A, Kela H, Saha ML, Maiti PK. Port-site infections by nontuberculous mycobacterium: a retrospective clinico-microbiological study. The International Journal of Mycobacteriology. 2017;6(1):34-7.

25. Durão V, Silva A, Macedo R, Durão P, Santos-Silva A, Duarte R. Portuguese in vitro antibiotic susceptibilities favor current nontuberculous mycobacteria treatment guidelines. Pulmonology. 2019;25(3):162-7.

26. Chew KL, Cheng JW, Hudaa Osman N, Lin RT, Teo JW. Predominance of clarithromycin-susceptible Mycobacterium massiliense subspecies: characterization of the Mycobacterium abscessus complex at a tertiary acute care hospital. Journal of Medical Microbiology. 2017;66(10):1443-7.

27. Fu L, Li GL, Hui P, Liu HC, Xiao TY, Li SJ, et al. Preliminary study on drug susceptibility profile and resistance mechanisms to macrolides of clinical isolates of non-tuberculous mycobacteria from China. Biomedical and environmental sciences. 2018;31(4):290-9.

28. Khosravi AD, Mirsaeidi M, Farahani A, Tabandeh MR, Mohajeri P, Shoja S, et al. Prevalence of nontuberculous mycobacteria and high efficacy of D-cycloserine and its synergistic effect with clarithromycin against Mycobacterium fortuitum and Mycobacterium abscessus. Infection and drug resistance. 2018:2521-32.

29. Lei Q, Wang H, Lv X, Dang L. Prevalence, Strains, and Drug Susceptibility of Nontuberculous Mycobacteria Isolates from Tuberculous Suspects. Jundishapur Journal of Microbiology. 2021;14(7).

30. Shipitsyna E, Unemo M. A profile of the FDA-approved and CE/IVD-marked Aptima Mycoplasma genitalium assay (Hologic) and key priorities in the management of M. genitalium infections. Expert Review of Molecular Diagnostics. 2020;20(11):1063-74.

31. Park J, Cho J, Lee C-H, Han SK, Yim J-J. Progression and treatment outcomes of lung disease caused by Mycobacterium abscessus and Mycobacterium massiliense. Clinical Infectious Diseases. 2017;64(3):301-8.

32. Garcia de Carvalho NF, Sato DN, Pavan FR, Ferrazoli L, Chimara E. Resazurin microtiter assay for clarithromycin susceptibility testing of clinical isolates of Mycobacterium abscessus group. Journal of Clinical Laboratory Analysis. 2016;30(5):751-5.

33. Asakura T, Suzuki S, Fukano H, Okamori S, Kusumoto T, Uwamino Y, et al., editors. Sitafloxacin-containing regimen for the treatment of refractory Mycobacterium avium complex lung disease. Open forum infectious diseases; 2019: Oxford University Press US.

34. Ruedas-López A, Tato M, Broncano-Lavado A, Esteban J, Ruiz-Serrano MJ, Sánchez-Cueto M, et al. Subspecies distribution and antimicrobial susceptibility testing of Mycobacterium abscessus clinical isolates in Madrid, Spain: a retrospective multicenter study. Microbiology Spectrum. 2023;11(3):e05041-22.

35. Watanabe J, Ihara H, Takei S, Nakamura A, Fujimoto Y, Handoh T, et al. The synergetic effect of sitafloxacin–arbekacin combination in the Mycobacterium abscessus species. Scientific Reports. 2023;13(1):2027.

36. Cheng A, Tsai Y-T, Chang S-Y, Sun H-Y, Wu U-I, Sheng W-H, et al. In vitro synergism of rifabutin with clarithromycin, imipenem, and tigecycline against the Mycobacterium abscessus complex. Antimicrobial agents and chemotherapy. 2019;63(4):10.1128/aac. 02234-18.

37. Fujiwara K, Aono A, Asami T, Morimoto K, Kamada K, Morishige Y, et al. In Vitro Synergistic Effects of Omadacycline with Other Antimicrobial Agents against Mycobacterium abscessus. Antimicrobial agents and chemotherapy. 2023;67(6):e01579-22.
